# Decomposing the gap in under-five mortality determinants between the low- and high-risk regions of Nigeria

**DOI:** 10.1038/s41598-025-20365-3

**Published:** 2025-10-17

**Authors:** Sunday A. Adedini, Elhakim Adekunle Ibrahim, Hassan Seun Ogunwemimo, Oluwarotimi Samuel Oladele, Sunday Matthew Abatan, Paul O. Adekola

**Affiliations:** 1 Demography and Social Statistics Department, Faculty of Social Sciences, Federal University Oye-Ekiti, Oye-Ekiti, Ekiti State Nigeria; 2 FUOYE Population Health Initiatives, Federal University Oye-Ekiti, Oye-Ekiti, Ekiti State Nigeria; 3 Programme in Demography and Population Studies, Schools of Public Health and Social Sciences, University of the Witwatersrand, Johannesburg, South Africa; 4 Department of Public Health, College for Health, Community and Policy, University of Texas at San Antonio, San Antonio, USA; 5 Centre for Data Science and Health Metrics, University of Medical Sciences, Ondo City, Nigeria; 6 Department of Population, Family and Reproductive Health, School of Public Health, University of Medical Sciences, Ondo City, Nigeria

**Keywords:** Under-five mortality, Low-risk regions, High-risk regions, Decomposition analysis, Nigeria, Health care, Risk factors

## Abstract

Nigeria has the highest burden of under-five mortality (U5M) in the world and contributes 17% of the global childhood deaths. The high national average of U5M in Nigeria is driven by the rates in some regions of the country. This study quantifies the contributions of socioeconomic, bio-demographic, and health-related factors in explaining the gaps in U5M between the country’s low-risk regions (LRR) and high-risk regions (HRR). The study performed spatial analysis (using Local Moran’s I spatial analysis) and Blinder-Oaxaca decomposition analysis of the gap in U5M determinants between the LRR and HRR on a sample of children born five years before the 2018 Nigeria Demographic and Health Survey (*n* = 32,234). Spatial analysis shows that the North-west and parts of the North-east regions were hotspots for U5M, while the South-west and parts of the South-east were coldspots. Results from the decomposition analysis revealed a prevalence of 6.1% in LRR compared with 11.3% in HRR. This translates to 58.9% higher U5M in HRR relative to LRR. The study concludes that interventions aimed at enhancing factors like maternal education, access to economic resources, family planning uptake, and increasing birth intervals in HRR could be effective in reducing high U5M in Nigeria.

## Introduction

Child mortality has reduced significantly across the world, with almost 60% decline from around 100 deaths per 1000 live births in 1990 to 37/1000 in 2022^1^. This translates to around 1 in 10 children dying before age five in 1990, compared to 1 in 27 in 2022. This development demonstrates better survival chances for children in recent years. In absolute terms, the total number of global under-five deaths declined from 12.8 million in 1990 to 4.9 million in 2022. However, the reduction in under-five mortality has been uneven across the world as children continue to face differing risks of death based on where they are born or raised. For instance, the two regions with the highest risks and burdens of under-five mortality – sub-Saharan Africa and southern Asia– account for more than 80% of the global under-five deaths in 2022^2^.

The gains recorded in child survival globally have stalled considerably in recent times. Evidence shows that progress in reducing under-five mortality during the post-2015 era of the Sustainable Development Goals (SDGs) has been much sluggish and slower compared to the 2000–2015 period of the Millennium Development Goals (MDGs). Recent data shows that the annual reduction rate in the global under-five mortality declined from 3.8% in 2000–2015 to 2.1% in the recent period, 2015–2022^2^. Also, with the increasing gaps in progress towards attaining the SDG target 3.2, which aims for reduction of under-five mortality rate (U5MR) to at least 25 deaths per 1,000 live births across the world’s regions, 59 countries, mostly of the sub-Saharan African region, may fall short of meeting the target by 2030^2^.

A recent study assessing child survival gains in Africa and the prospects for achieving the SDG target on child mortality found that, out of the 54 African countries, only 8 have met the SDG target 3.2^3^. Many countries may not reach this target without appropriate investments and interventions. Furthermore, the study shows that 7 of the 54 African countries (Nigeria, Niger, Togo, Burkina Faso, Cameroon, Chad) are hotspots for under-five mortality, with Nigeria having the highest under-five mortality rate among these 7 countries^3^.

Although Nigeria has seen some reductions in childhood deaths, it still carries the highest burden of under-five mortality worldwide. Statistics show that Nigeria contributed 835,030 deaths among children aged 0 to 59 months in 2022, representing 17% of global under-five deaths^4^. Meanwhile, theNigeria’s under-five mortality has huge sub-national variations, with the rate higher in some states and regions than in others^12^. For instance, the recently released key indicators report for the 2023-24 Nigeria Demographic and Health Survey (NDHS) shows that the under-five mortality rate is lowest in the South West (42 deaths per 1,000 live births) and highest in the North West (140 deaths per 1,000 live births)^13^. The report further indicates intra-regional variations in childhood mortality rates. For the South West States, the under-five mortality rate is lowest at 15 deaths per 1,000 live births in Ondo State and highest at 60 deaths per 1,000 live births in Ogun State. Further evidence from the key indicators report indicates that the child mortality rate (4q1) per 1000 live births is lowest in Oyo State (5 deaths per 1,000 live birth) and highest in Ekiti State (18 deaths per 1,000 live births).

Studies have established that the drivers of high under-five mortality in Nigeria are diverse^5^. These include limited access to quality child healthcare^6^, delays in seeking care^7^, high-risk status at birth^8^, high unmet need for family planning^9,10^, as well as cultural, maternal and environmental factors^11^. For example, poor access to essential maternal and child healthcare services during prenatal and postnatal periods significantly elevates the risk of dying among under-five children, particularly in low-resource settings where critical health interventions are scarce^6^. The risk can be further compounded by delays in seeking needed medical care due to low health literacy, financial constraints, cultural impediments, or geographic barriers^7^. Risky childbearing practices, including too early/late childbirth, too close subsequent births, and grand-multiparity, undermine maternal and child health and survival outcomes^8^. Besides, environmental factors such as household poverty, poor sanitation, and inadequate access to clean water contribute to child mortality by increasing exposure to poor nutrition and infectious diseases while limiting access to timely medical intervention^11^. Addressing these gaps requires targeted interventions to strengthen national healthcare systems and improve maternal-child health outcomes.

Evidently, the high national average in childhood mortality in Nigeria is largely driven by the disproportionately high rate of child deaths in some regions and states in the country. Nigeria will significantly reduce childhood mortality if targeted interventions are implemented in high-mortality regions of the country. This raises the need to decompose the gap in under-five mortality determinants between Nigeria’s low- and high-risk regions. Besides, understanding disparities and the factors sustaining under-five deaths in high-mortality regions is critical for framing appropriate interventions. Evidence is sparse on this. Hence, this study aims to provide evidence on the factors sustaining high under-five mortality in the high-risk regions of Nigeria. The study quantified the contributions of socioeconomic, bio-demographic, and health-related factors in explaining the gap in under-five mortality between the country’s low - and high-risk regions.

## Data and methods

### Data source

The study used cross-sectional secondary data from the 2018 Nigeria Demographic and Health Survey. The study relied on the survey’s birth-recode data set, using the child born within the five years before the survey as the unit of analysis. The NDHS elicits demographic and health information from nationally representative samples using a stratified two-stage cluster design sampling technique. Enumeration areas (EAs) were the primary sampling unit, while a whole listing of households was done in selected EAs to derive representative samples across Nigeria. Structured questionnaires were used to elicit information. A significant part of the questionnaires used covered childbearing experience, which involved asking women about their birth histories, including the number of children ever-born and the number of children dead and alive. Data on birth history were recoded as separate birth-recode datasets for children aged 0–59 months, including information on their date of birth, current age for living children, or age at death for dead children, and other relevant background characteristics.

### Variable measurements

#### Outcome variable

The outcome variable considered in this study was under-five death, defined as the risk of a live-born child dying before their fifth birthday. The variable was operationalised as a dichotomous variable assigned the value of 1 if the child died before the reference age of 5, and 0 if the child survived past the age of 5^14^.

#### Explanatory variables

Relevant explanatory variables were selected for the study with the guidance of the literature. We identified key drivers of under-five mortality that were available in the 2018 NDHS dataset based on findings from previous studies. The selected variables and their operational definitions are presented in Table 1.

**Table 1:** Independent variables for modelling children’s survival chances in the high- and low-risk regions of Nigeria.

Variables	Operational definitio*
Child’s sex	Sex of the child: male vs. female
Child’ birth size	Size of the child at birth: larger birth size vs. average birth size, smaller birth size
Child’s birth order	The child’s birth position or order: first birth order vs. birth order 2–3, birth order 4+
Child’s birth interval	Child’s inter-birth interval in months: <36 months vs. 36–59 months, 60 + months
Age at child’s birth	Maternal age at child’s birth in years: <20 years vs. 20–34 years, birth age 35 + years
Current marital status	Mother’s marital status at the time of the survey: currently married vs. not currently married
Religious affiliation	Mother’s religious affiliation: Islamic religion vs. Non-Islamic religion
Occupational category	Maternal occupation at the time of the survey: no occupation vs. formal occupation, informal occupation
Family planning status	Contraceptive use by child’s mother: ever used family planning vs. never used family planning
Mass media exposure	Exposure of child’s mother to media content: has media exposure vs. has no media exposure
Educational status	Maternal highest level of education attained: no education vs. primary education, secondary + education
Family structure	Type of family structure: monogamous family vs. non-monogamous family,
Household headship	Sex of the household head: male-headed household vs. female-headed household
Household wealth quintile	The composite index of household items: lower quintile household vs. middle quintile household, upper quintile household
No. of household members	Number of persons living in the household: less than five members vs. at least five members,
Type of drinking water	Source of water used for drinking: safe drinking water vs. unsafe drinking water
Type of toilet facility	Type of toilet facility: Safe toilet facility vs. unsafe toilet facility
Type of cooking fuel	Type of energy used for coking: safe cooking fuel vs. unsafe cooking fuel
Barrier to health care	Challenges encountered while seeking health care: experienced barrier vs. experienced no barrier
Residential setting	Residential setting: rural vs. urban

*The first category of each variable considered as the reference group.

### Statistical analysis

Analyses for this study were conducted using the open-source QGIS for Desktop version 3.38 Grenoble (accessible at https://qgis.org/project/ from QGIS Association)^46^ and the Stata/SE Statistical Software version 14.2 (accessible at https://www.stata.com/stata14/ from StataCorp LP, College Station, Texas, USA)^47^. In line with the aim of the study, the estimates of under-five mortality per 1000 live births were extracted from the DHS report by state and spatial distribution, from which a Local Moran’s I clustering map was generated about the six administrative regions of Nigeria. These are classified as low-risk and high-risk regions of mortality among under-five children based on the prevalence rates contained in the 2018 NDHS report^13,45^. Nigeria is administratively divided into six geopolitical zones, comprising three regions, each located in the north and the south. Each region typically comprises an average of six states. Although the global under-five mortality rate has decreased by 60 per cent, dropping from about 100 deaths per 1,000 live births in the 1990 s to 37 in 2022, many regions and states in Nigeria still experience rates exceeding 100 deaths per 1,000 live births even after more than three decades since the 1990s. Accordingly, any Nigerian region with U5MR below 100 deaths per 1,000 live births was considered as a low-risk region while those with 100 deaths and above per 1,000 live births were designated as a high-risk region. The U5MR cut-off adopted was informed by its reference as a very high rate by the United Nations Inter-Agency Group for Child Mortality Estimation^42^. While many U5MR below 100 deaths per 1000 live births in Nigeria are still issues of concern, those above the 100 deaths per 1000 live births benchmark require even more urgent attention to begin to address the high deaths among children before their fifth birthday.

The study’s objective was achieved by presenting descriptive and inferential statistics. Accordingly, frequency and percentage distribution were employed to describe the background characteristics of the study population by regional classification of the level of under-five mortality. The population level pattern of U5MR was explored based on the mortality rates calculated using the full birth history method and estimation procedure of the DHS program as applied in various Demographic and Health Surveys reports, including the 2018 NDHS. The study further leveraged spatial analytics technique anchored on Local Moran’s I statistics to assess the inter- and intra-regional dynamics of under-five mortality rates in the Nigerian context. As an approach for exploring spatial autocorrelation and clustering of key sociodemographic and health events, the Local Moran’s I statistics help to identify statistically significant hotspots (high-high) and coldspots (low-low), revealing the localised patterns of events of interest across geographic units as clusters and outliers^15^.

Data for spatial analysis were aggregated at the state level, and other attributes of the aggregated data are labels of both states and regions. The Local Moran’s I analysis, conducted at three levels, was first carried out among states in the high U5M regions, followed by those in the low U5M regions. Spatial analysis was then conducted at the national level using state-based aggregated U5MR. In efforts to reduce aggregation bias in the maps produced, both the Queens Contiguity Method and Sensitivity Analysis were employed. Sensitivity analyses conducted explored a range of scaling recommended for aggregated data at both 1:500000 and 1:1000000. In both cases, there were no marked differences in the results. This approach, largely aimed at fostering targeted interventions, has been widely used in literature to identify hotspots of diverse health outcomes^40,41^.

Consequent on the results, areas with no spatial autocorrelation significance were areas typically with U5MR which were randomly distributed, and no clustering or outlier was observed. High-High areas - the hotspot areas - with high U5MR surrounded by other areas also with high U5MR. In contrast, Low-Low areas were coldspot areas with low U5MR and surrounded by other low U5MR areas. These were clusters of low U5MR. The other two categories – High-Low and Low-High – were outliers representing areas with high U5MR surrounded by those with Low U5MR and areas with low U5MR surrounded by those with high U5MR, respectively. These are illustrated in Figs. 2, 3 and 4.

**Fig. 1:**
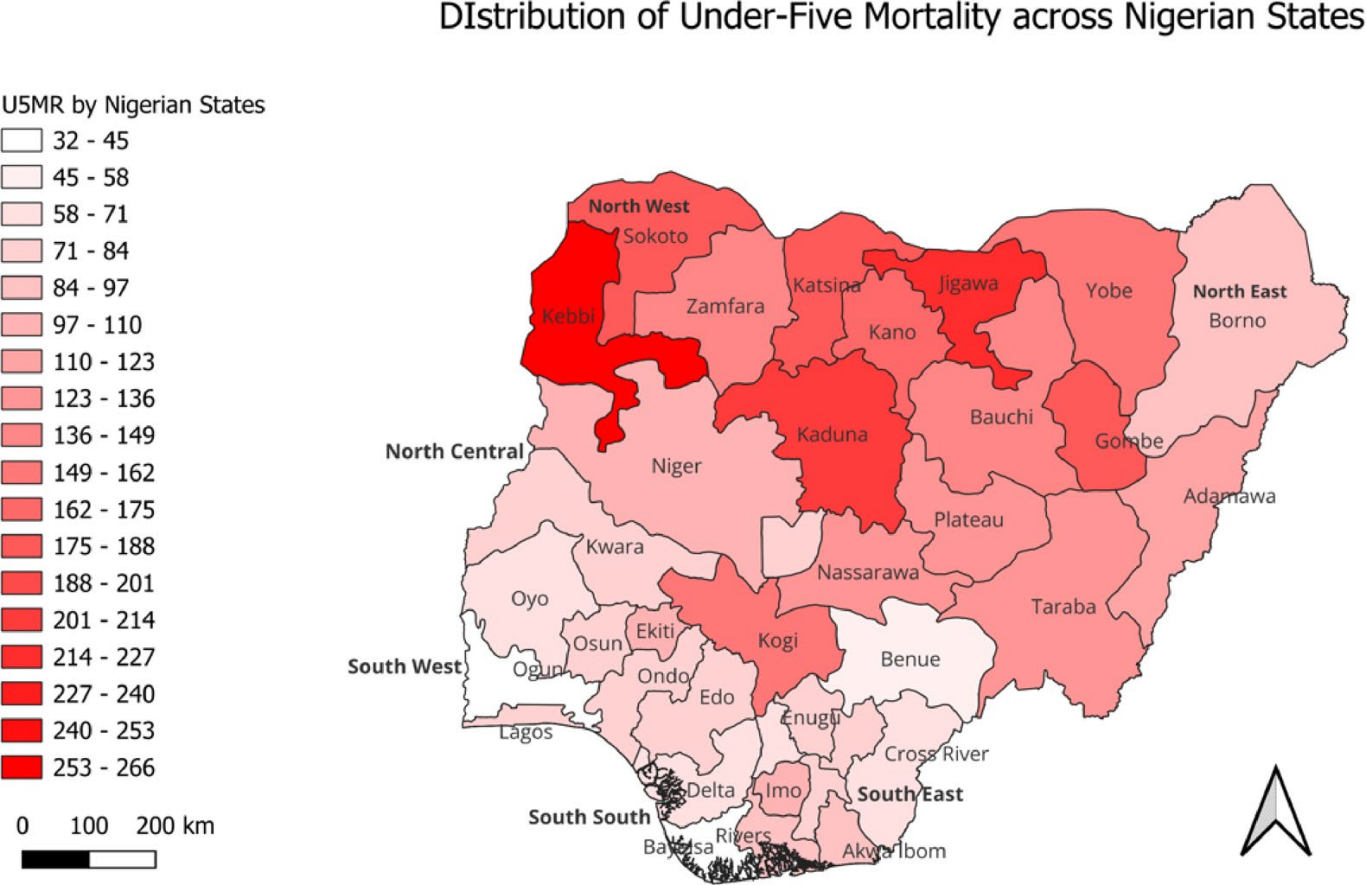
Distribution of U5MR/1000 live births across Nigerian states

**Fig. 2:**
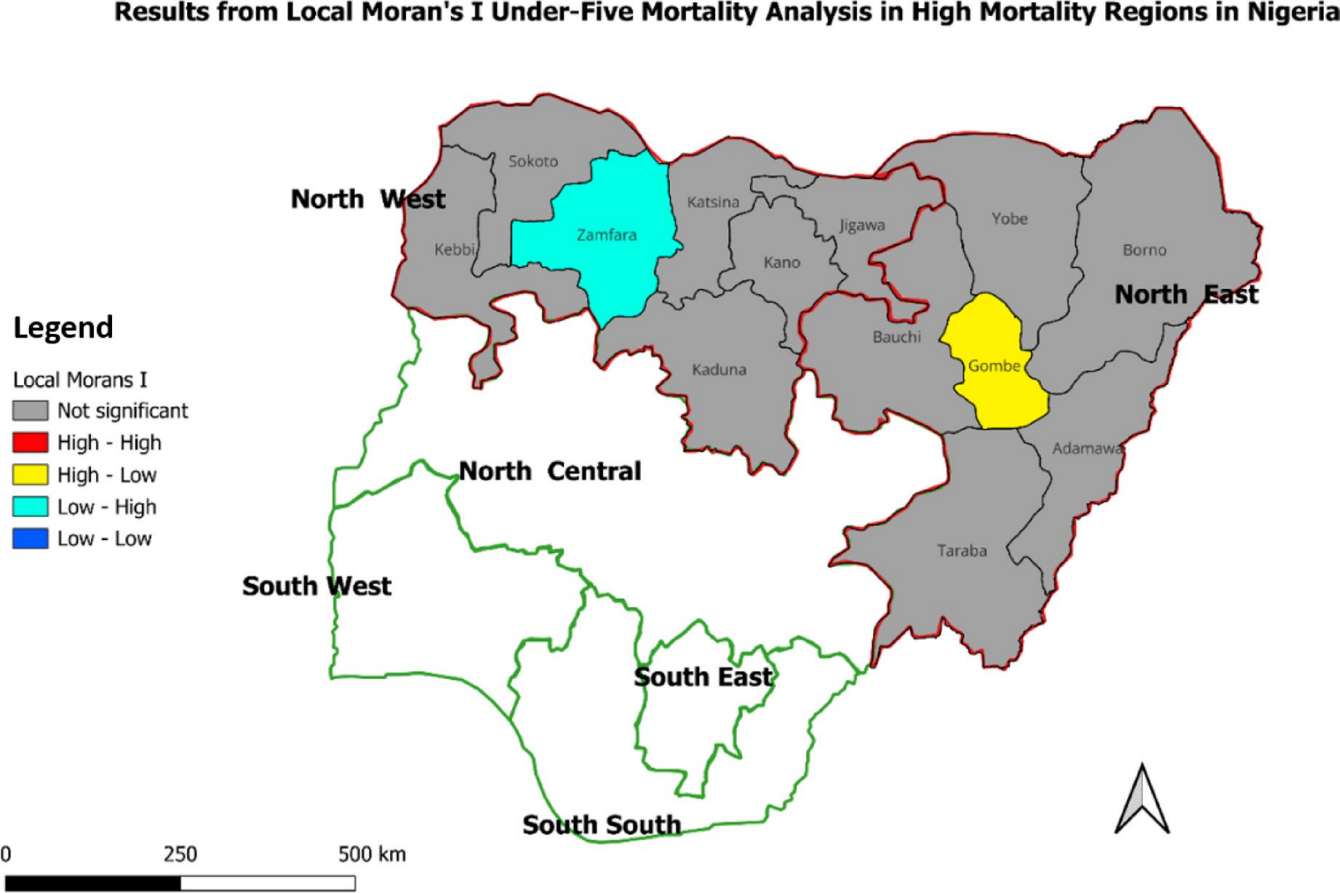
Results from Local Moran’s I analysis in Nigeria’s high U5M regions.

**Fig. 3:**
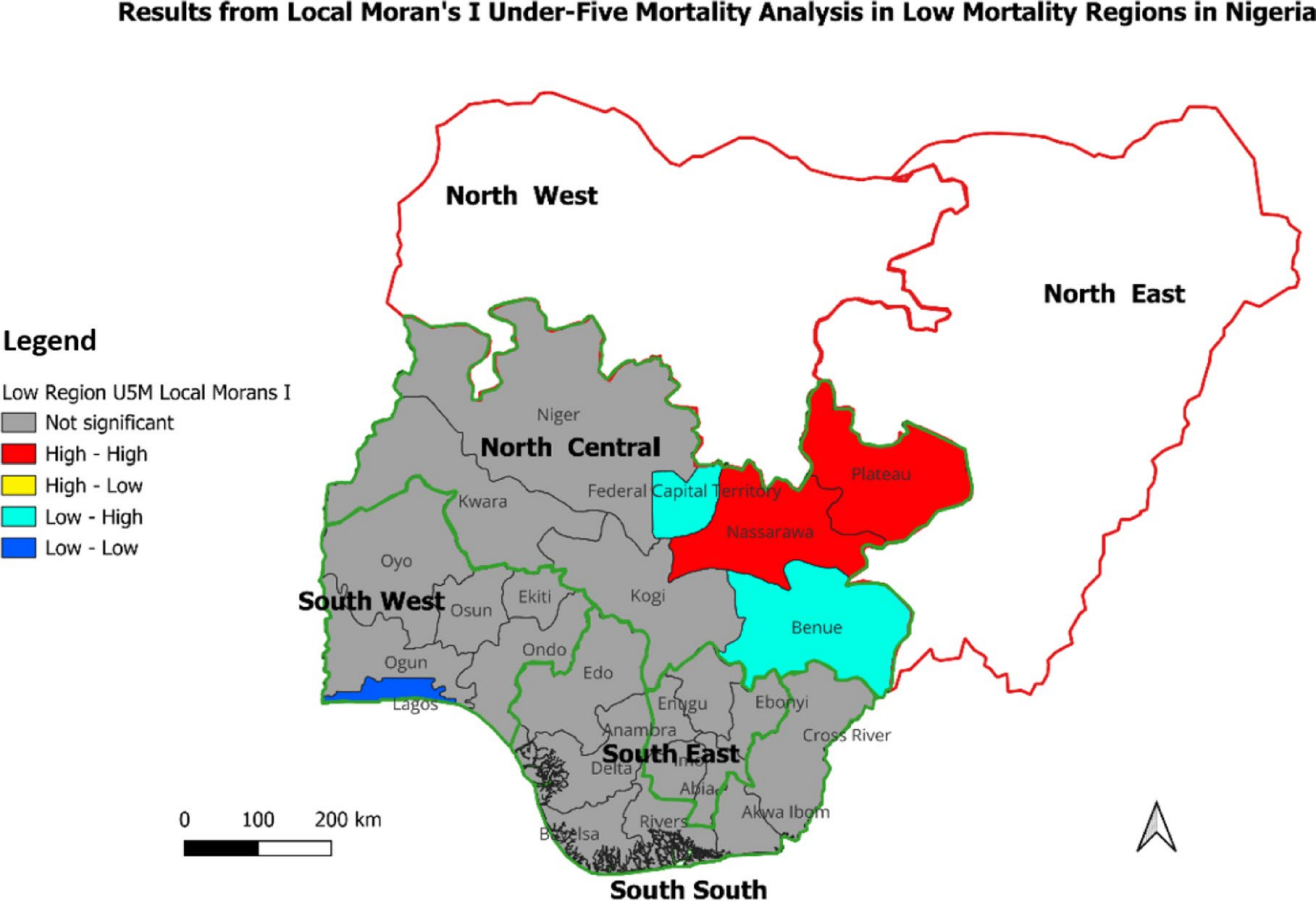
Results from Local Moran’s I analysis in Nigeria’s low U5M regions.

**Fig. 4:**
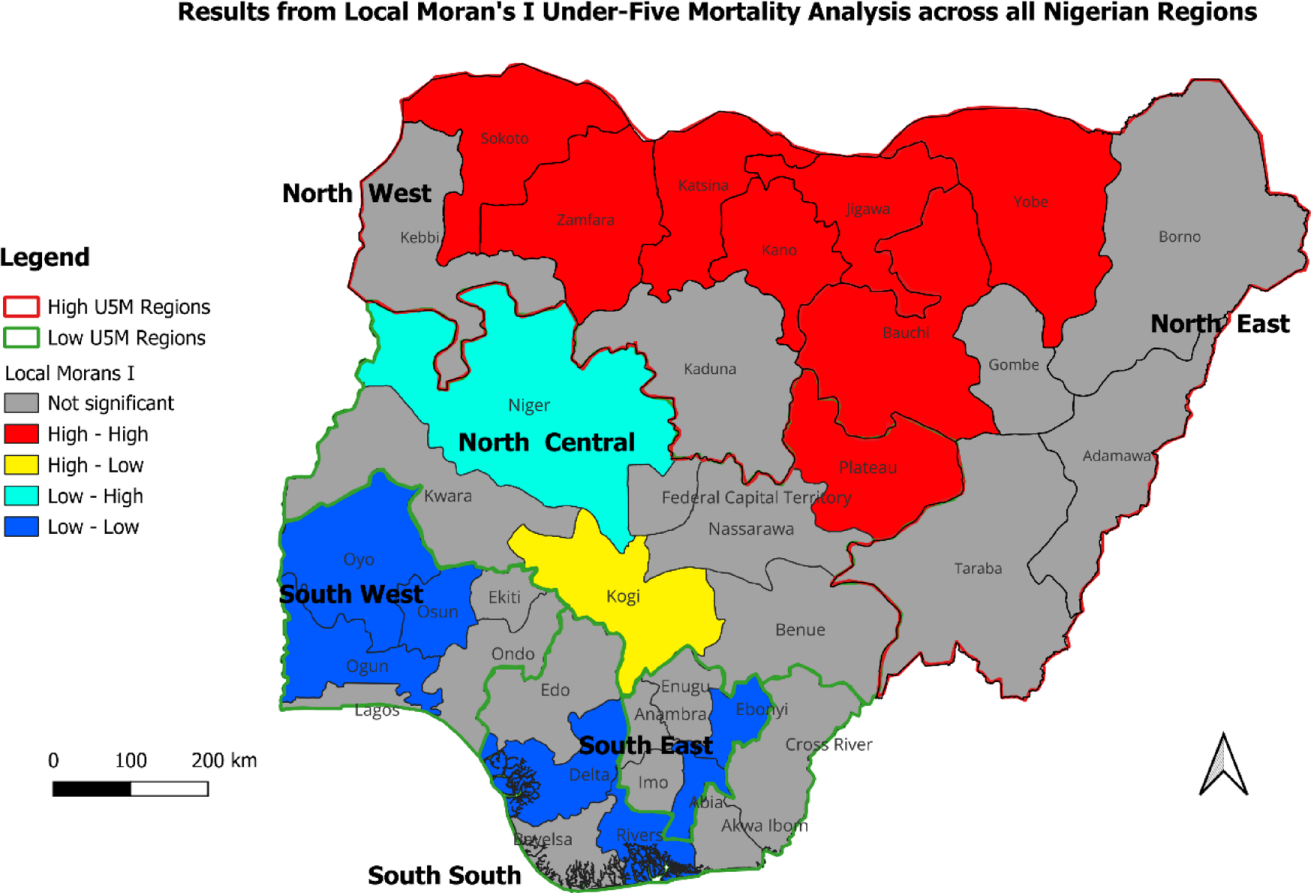
Results from Local Moran’s I analysis across Nigerian states (all regions).

Additionally, a multivariate logistic decomposition analysis was performed using the Blinder-Oaxaca decomposition method^16^. This method allows for quantifying the child survival gap between the “advantaged” and the “disadvantaged” groups by estimating the contribution of observed predictors to under-five mortality risk differences between the high- and low-risk regions. The decomposition technique estimates group disparities by decomposing the risk difference into its explained (the endowment effects - E) and unexplained (the coefficient effects - C) components. The endowment effects component reflects the disparity attributable to differences in the levels of the observed characteristics, while the coefficient effects indicate the gap due to differences in the effect of the coefficients of the determinants (the coefficient effects - C). Detailed explanation of this procedure and its variants is contained in published studies^16,21,23,25,26^.

### Ethical clearance

The study used a publicly available secondary dataset from the Demographic and Health Survey for which ethical clearance was already obtained and all unique identifiers removed. Permission to use the data for this study was formally obtained from the ICF International, USA.

## Results

### Descriptive analysis

The background characteristics of the respondents are presented in Table 2. The table highlights the differences in socio-economic characteristics between the low-risk and high-risk regions, with factors such as education, wealth index, access to safe drinking water, and media exposure contributing to lower child mortality in low-risk regions. The table presents differences in maternal and child characteristics between the low-risk and high-risk regions. As shown in Table 2, early motherhood (18.1%), higher-order births (54.7%), short birth intervals (21.9%), and use of unsafe cooking fuel (97.8%) were moreprevalent in high-risk than in low-risk regions. Conversely, first-order births (24%), media exposure (79.8%), women with at least secondary education (65.4%), households at higher wealth quintile (56.4%), and households with improved drinking water and toilet facilities were more common in low-risk regions compared to high-risk regions.

**Table 2:** Percentage distribution of characteristics of the study sample by regional classification – low-risk and high-risk regions in Nigeria.

Characteristics	Under-five children				% Difference
	LRR = 14,410		HRR = 17,824		
	Count	Percent	Count	Percent	
Age at childbirth					
Birth age < 20 yrs	1277	8.9	3,234	18.1	9.2
Birth age 20–34 yrs	10,960	76.1	11,951	67.0	−9.1
Birth age 35 + yrs	2173	15.1	2,639	14.8	−0.3
Marital status					
Currently married	13,477	93.5	17,276	96.9	3.4
Never/once married	933	6.5	548	3.1	−3.4
Child’s birth order					
First birth order	3459	24.0	2,908	16.3	−7.7
Birth order 2–3 pos	5729	39.8	5,163	29.0	−10.8
Birth order 4 + pos	5221	36.2	9,753	54.7	18.5
Child’s birth interval					
First birth order	3459	24.0	2,908	16.3	−7.7
Birth interval < 24 mos	2548	17.7	3,899	21.9	4.2
Birth interval 24–35 mos	3936	27.3	6,014	33.7	6.4
Birth interval 36 + mos	4467	31.0	5,004	28.1	−2.9
Child’s sex					
Child is male	7401	51.4	9,041	50.7	−0.7
Child is female	7008	48.6	8,783	49.3	0.7
Child’s birth size					
Larger birth size	4381	30.4	6,391	35.9	5.5
Average birth size	8146	56.5	8,620	48.4	−8.1
Smaller birth size	1882	13.1	2,812	15.8	2.7
Religious affiliation					
Islamic religion	4153	28.8	16,457	92.3	63.5
Non-Islamic religion	10,256	71.2	1,367	7.7	−63.5
Mass media exposure					
Has media exposure	11,492	79.8	8,170	45.8	−34
Has no media exposure	2917	20.2	9,654	54.2	34
Occupational category					
No occupation	2376	16.5	6,885	38.6	22.1
Formal occupation	1358	9.4	439	2.5	−6.9
Informal occupation	1067	74.1	10,500	58.9	−15.2
Educational status					
No education	3773	16.5	12,681	71.1	54.6
Primary education	2619	18.2	2,213	12.4	−5.8
Secondary + education	9418	65.4	2,930	16.4	−49
Family planning status					
Ever used family planning	6852	47.6	3,180	17.8	−29.8
Never used family planning	7557	52.4	14,644	82.2	29.8
Household wealth quintile					
Lower quintile household	3164	22.0	11,396	63.9	41.9
Middle quintile household	3116	21.6	3,482	19.5	−2.1
Upper quintile household	8129	56.4	2,946	16.5	−39.9
Family structure					
Monogamous family	11,386	79.0	9,761	54.8	−24.2
Non-monogamous family	3023	21.0	8,063	45.2	24.2
No. of household members					
Below five members	5118	35.5	3,665	20.6	−14.9
At least five members	9292	64.5	14,159	79.4	14.9
Type of drinking water					
Safe drinking water	9731	67.5	9,489	53.2	−14.3
Unsafe drinking water	4678	32.5	8,334	46.8	14.3
Type of toilet facility					
Safe toilet facility	8458	58.7	8,103	45.5	−13.2
Unsafe toilet facility	5951	41.3	9,721	54.5	13.2
Type of cooking fuel					
Safe cooking fuel	2593	18.0	394	2.2	−15.8
Unsafe cooking fuel	11,816	82.0	17,430	97.8	15.8
Barrier to health care					
Experienced barrier	7474	51.9	9,956	55.9	4
Experienced no barrier	6935	48.1	7,868	44.1	−4
Residential setting					
Rural residential setting	6401	44.4	13,437	75.4	31
Urban residential setting	8008	55.6	4,386	24.6	−31
Under-five mortality					
Alive	13,531	93.9	15,810	88.7	−5.2
Dead	879	6.1	2014	11.3	5.2

*LRR* Low-risk regions, *HHR* High-risk regions.

#### Under-five mortality rates between high-risk and low-risk regions

On average, the rate of under-five deaths was more than double in high-risk regions (172 deaths per 1,000 live births) compared to low-risk regions (82 deaths per 1,000 live births). The rates of under-five mortality by selected characteristics are presented in Table 3.

**Table 3:** Rates of under-five mortality by regional classification of under-five mortality risk in Nigeria.

	Low-risk regions		High-risk regions		All regions	
	Rate	95% CI	Rate	95% CI	Rate	95% CI
	82	76–88	172	161–183	132	125–139
Child’s sex						
Child is male	89	81–97	176	163–189	137	129–145
Child is female	75	68–83	168	153–184	127	118–137
Child’s birth size						
Larger birth size	71	59–84	147	132–163	116	106–127
Average birth size	83	74–93	170	153–189	128	117–139
Smaller birth size	116	101–132	209	188–231	169	154–184
Child’s birth order						
First birth order	70	61–80	170	150–193	116	104–128
Birth order 2–3 pos	71	63–79	163	147–180	115	106–125
Birth order 4 + pos	103	92–114	177	166–189	151	143–160
Child’s birth interval						
First birth order	70	61–80	176	154–200	118	106–132
Birth interval < 24 mos	105	91–120	228	208–250	183	169–199
Birth interval 24–35 mos	81	73–91	168	155–180	134	125–143
Birth interval 36 + mos	79	66–93	116	105–129	97	88–106
Child’s birth interval						
First birth order	70	61–80	176	154–200	118	106–132
Birth interval < 36 mos	91	82–100	193	180–207	154	145–164
Birth interval 36–59 mos	82	66–103	120	108–134	102	92–114
Birth interval 60 + mos	70	57–87	98	78–123	81	69–94
Age at child’s birth						
Birth age < 20 yrs	82	69–98	189	169–210	159	144–175
Birth age 20–34 yrs	80	74–87	167	155–179	126	118–133
Birth age 35 + yrs	91	77–108	174	157–193	137	125–150
Age at first marriage						
Age at marriage < 18 yrs	95	84–108	180	169–192	160	151–170
Age at marriage 18 + yrs	77	71–83	145	123–170	97	88–106
Current marital status						
Currently married	80	74–86	173	162–184	132	125–140
Not currently married	112	83–149	149	117–188	126	104–152
Religious affiliation						
Islamic religion	69	58–82	110	71–166	71	60–84
Non-Islamic religion	85	78–92	172	162–184	137	130–145
Occupational category						
No occupation	90	76–105	174	161–187	155	144–166
Formal occupation	68	54–86	140	108–180	87	73–104
Informal occupation	83	76–90	172	159–185	127	119–135
Family planning status						
Ever used family planning	67	60–74	121	108–135	84	77–91
Never used family planning	97	87–107	183	172–196	155	146–164
Mass media exposure						
Has media exposure	76	70–83	154	142–167	109	102–116
Has no media exposure	104	92–118	187	173–201	167	156–179
Educational status						
No education	97	83–113	191	179–204	176	164–187
Primary education	96	80–113	160	135–190	125	110–141
Secondary + education	73	66–81	88	74–104	76	70–83
Family structure						
Monogamous family	75	69–82	159	147–173	114	107–122
Non-monogamous family	105	91–121	185	172–200	163	152–175
Household headship						
Male-headed household	81	75–87	173	162–184	135	127–142
Female-headed household	91	73–113	145	115–181	105	90–124
Household wealth quintile						
Lower quintile household	101	88–115	195	182–208	175	164–186
Middle quintile household	100	88–113	159	138–182	130	117–145
Upper quintile household	68	61–75	96	81–114	75	69–82
No. of household members						
Below five members	115	102–130	272	240–306	174	158–191
At least five members	66	60–73	153	144–163	119	112–126
Type of drinking water						
Safe drinking water	78	71–87	166	153–181	124	115–133
Unsafe drinking water	90	80–101	180	165–195	145	135–157
Type of toilet facility						
Safe toilet facility	74	66–83	149	134–166	111	102–121
Unsafe toilet facility	92	83–103	190	178–203	153	144–162
Type of cooking fuel						
Safe cooking fuel	64	55–75	68	40–112	64	56–75
Unsafe cooking fuel	92	85–100	175	164–186	146	138–155
Barrier to health care						
Experienced barrier	86	77–95	185	172–198	143	134–152
Experienced no barrier	78	70–87	155	142–169	119	111–128
Residential setting						
Rural residential setting	89	81–98	189	177–202	157	147–167
Urban residential setting	76	68–85	119	103–139	92	83–101

*CI* Confidence interval

### Spatial analysis

#### Distribution of U5MR/1000 live births across Nigerian states

This section presents spatial analysis across the 36 States of Nigeria. Descriptively and delineated by high- and low-U5MR regions or simply low- and high-risk regions, Fig. 1 shows the distribution of U5MR across Nigerian states. It revealed that the North East and the North West, particularly, were home to the highest rates of U5M in Nigeria. Except in Zamfara State (144 deaths/1,000 live births), U5MR was higher than 144 deaths per 1,000 live births in almost all the states in the latter region, and they included Kebbi, Sokoto, Katsina, Kano, Jigawa and Kaduna States. Also, except in Borno State (86 deaths/1,000 live births), U5MR was high in the North East, with most states, including Yobe, Gombe, Bauchi, Plateau, and Taraba states, having rates higher than 114 deaths per 1,000 live births. While states in the North Central had relatively lower U5MR compared to the majority of states in the North East and the North West, such rates were still high in some states in this region. Other than in Benue with the least U5MR in the region (52 deaths/1,000 live births) and in the Federal Capital Territory with 73 deaths/1,000 live births, states in the North Central region, including Kwara, Niger, Plateau and Kogi States, had U5MR ranging between 82 deaths/1,000 live births and 155 deaths/1,000 live births. Compared to other regions, the South West and South South regions had the lowest distribution of U5MR in Nigeria, with the former having states with the least U5MR distribution. Essentially, in the South West and indeed across Nigeria, Ogun State had the least U5MR (30 deaths/1,000 live births), while Ekiti State had the highest U5MR distribution in the South Western states (95 deaths/1,000 live births).

### Spatial analysis of intra-regional U5M clustering

#### High-risk/high U5M regions

##### Local Moran’s I report: intra-regional under-five mortality rates in high-risk/high mortality regions (North West and North East Nigeria)

As shown in Fig. 2, the majority of states in the North West region were coded brown, indicating that they did not exhibit statistically significant spatial clustering or outliers in U5MR based on the Local Moran’s I analysis. Zamfara was coded in turquoise blue, signifying that it was a Low-High outlier. This result implied that, despite being in a region known for high under-five mortality, Zamfara had a relatively low under-five mortality rate compared to its neighbouring states, which generally had higher rates. Similar to the North West, the majority of the states in the North East region were coded brown, indicating no significant spatial clustering or outliers. Gombe was coded Yellow, indicating it was a High-Low outlier with a higher under-five mortality rate compared to its neighbouring states. Summarily, when explored among themselves, the majority of states in the high U5M regions did not exhibit significant spatial clustering or outliers (coded brown). However, the identification of outliers such as Zamfara (Low-High) and Gombe (High-Low) provides critical insights into specific areas that either perform better or worse than their regional counterparts.

#### Low-risk/Low U5M regions

##### Local Moran’s I report: intra-regional under-five mortality rates in low-risk/low mortality regions (South West, South East, South-South, and North Central, Nigeria)

As shown in Fig. 3, the majority of states in the South West region were coded brown, indicating no statistically significant spatial clustering or outliers in U5MR. However, Lagos was coded blue, signifying it as a coldspot. This indicated that Lagos had a significantly lower under-five mortality rate compared to its neighbouring states, which relatively had higher rates. All states in both the South East and South South regions were coded brown, indicating that there were no statistically significant spatial clusters or outliers in under-five mortality rates. States in the North Central region appeared more diverse than those in other regions. While the Federal Capital Territory and Benue were coded turquoise blue, indicating their outlier status, where both were low U5MR states surrounded by high U5MR states, both Plateau and Nasarawa were coded red, indicating they were high U5MR states situated around other high U5MR states.

Summarily, the identification of Lagos as a coldspot highlighted it as a particularly successful state in reducing under-five mortality within the region. In contrast, both Plateau and Nasarawa were identified as hotspot states, stressing the need for special attention to addressing U5MR in these states. Also, the low-high outlier status of FCT Abuja and Benue suggests that these states were performing better than their neighbours in terms of U5MR, and the absence of significant clusters or outliers in the South East and the South South points to a uniform situation regarding under-five mortality rates across these states.

#### Local moran’s I report: inter-regional under-five mortality rates across all six regions in Nigeria

Figure 4 presents the spatial analysis of inter-regional variations in U5M across the 6 regions of Nigeria. Summarily, the North West and parts of the North East regions were clear hotspots for under-five mortality, with most states in these areas showing significantly higher rates. The South West, along with specific states in the South East (Abia, Enugu) and South South (Rivers, Delta), were coldspots, indicating lower under-five mortality rates. Notable outliers included Kogi (High-Low) and Niger States (Low-High) both in the North Central, highlighting areas with rates that deviate from regional trends.

### Decomposition analysis

**Fig. 5:**
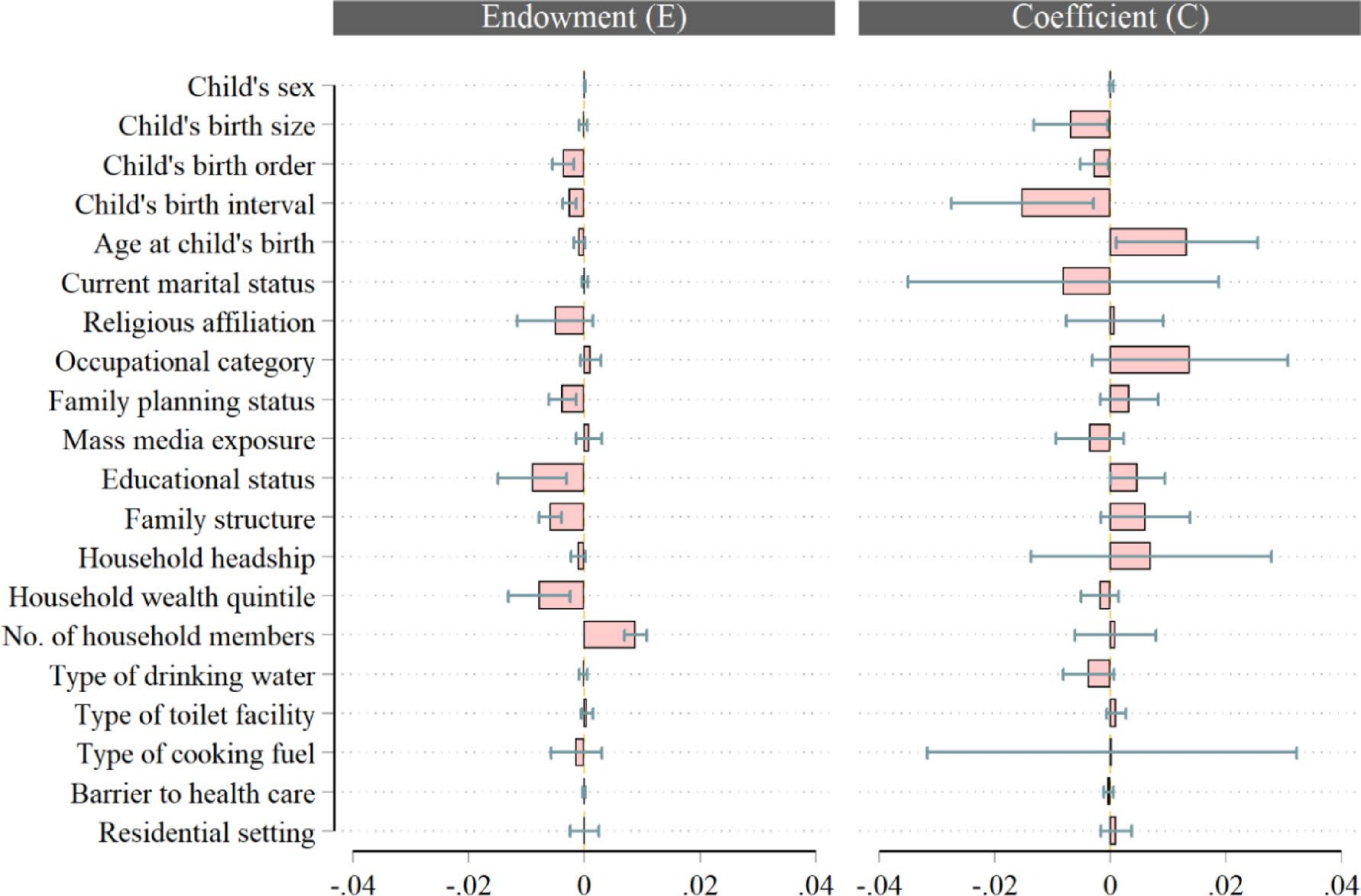
Chart showing the summary decomposition of the gap in under-five mortality risk between LRR and HRR and relative contributions of the observed predictors of under-five deaths to (un)explained components.

Tables 4, 5 and 6 present the decomposition of the regional differential in under-five mortality likelihoods between the low-risk regions (LRR) and high-risk regions (HRR) in Nigeria. The analysis decomposed the gap into explained and unexplained components. In the summary results presented in Table 4, the differential subsection shows the average prevalence of under-five mortality in the LRR and HRR, and the gap between the groups. The results revealed a prevalence of about 6.1% in LRR compared with 11.3% in HRR, culminating in approximately a 5.1%-point significant difference between the groups. In relative terms, this gap in prevalence rates further translates to about 58.9% higher under-five deaths in HRR than LRR, consistent with the results in Fig. 5 depicting considerably greater risks of under-five deaths in the HRR relative to the LRR.

**Table 4:** Summary decomposition of the gap in under-five mortality risk between LRR and HRR in Nigeria.

	Coef.	Global estimates	*P*-value	Pct.
		95% CI		
Differential:				
Low-risk regions average prevalence	0.06134	0.05662–0.06606	0.000	-
High-risk regions average prevalence	0.11260	0.10564–0.11957	0.000	-
Raw difference in average prevalence	−0.05126	−0.05968–0.04285	0.000	-
Decomposition:				
Explained component (Endowment - E)	−0.03067	−0.04057–0.02077	0.000	59.8
Unexplained component (Coefficient - C)	−0.02059	−0.03309–0.00809	0.001	40.2

Coef., cluster-adjusted coefficients estimated by pooled Oaxaca-Blinder decomposition (including a mortality region indicator). *CI* Confidence interval. Pct., relative percentage of raw difference attributable to decomposition component.

*LRR* Low-risk region, *HRR* High-risk region

**Table 5:** Summary decomposition of the gap in under-five mortality risk between LRR and HRR in Nigeria by specific predictors of under-five deaths.

	Explained component (Endowment - E)				Unexplained component (Coefficient - C)			
	Coef.	95% CI	*P*-value	Pct.	Coef.	95% CI	*P*-value	Pct.
Child’s sex	0.00005	−0.00006-0.00016	0.402	−0.2	0.00015	−0.00016-0.00046	0.339	−0.7
Child’s birth size	−0.00022	−0.00093-0.00049	0.544	0.7	−0.00682	−0.01317–0.00048	0.035	33.1
Child’s birth order	−0.00476	−0.00682–0.0027	0.000	15.5	0.00316	−0.00250-0.00881	0.274	−15.3
Child’s birth interval	−0.00155	−0.00234–0.00076	0.000	5.1	−0.02831	−0.04809–0.00853	0.005	137.5
Age at child’s birth	−0.00093	−0.00190-0.00004	0.061	3.0	0.01325	0.00105–0.02545	0.033	−64.3
Current marital status	0.00005	−0.00048-0.00058	0.847	−0.2	−0.00815	−0.03505-0.01875	0.553	39.6
Religious affiliation	−0.00507	−0.01164-0.0015	0.130	16.5	0.00078	−0.00766-0.00922	0.856	−3.8
Occupational category	0.00107	−0.00073-0.00288	0.245	−3.5	0.01381	−0.00315-0.03076	0.111	−67.0
Family planning status	−0.00384	−0.00623–0.00145	0.002	12.5	0.00329	−0.00170-0.00828	0.196	−16.0
Mass media exposure	0.00079	−0.00140-0.00298	0.482	−2.6	−0.00353	−0.00942-0.00237	0.241	17.1
Educational status	−0.00903	−0.01491–0.00314	0.003	29.4	0.00473	0.00000-0.00945	0.050	−22.9
Family structure	−0.00589	−0.00777–0.00401	0.000	19.2	0.00608	−0.00161-0.01377	0.121	−29.5
Household headship	−0.00106	−0.00234-0.00022	0.106	3.5	0.00704	−0.01378-0.02786	0.507	−34.2
Household wealth quintile	−0.00788	−0.01322–0.00254	0.004	25.7	−0.00181	−0.00509-0.00147	0.280	8.8
No. of household members	0.00885	0.00699–0.01071	0.000	−28.9	0.00088	−0.00609-0.00785	0.805	−4.3
Type of drinking water	−0.00025	−0.00094-0.00045	0.486	0.8	−0.00378	−0.00818-0.00062	0.092	18.4
Type of toilet facility	0.00045	−0.00062-0.00152	0.412	−1.5	0.00102	−0.00066-0.00270	0.236	−4.9
Type of cooking fuel	−0.00141	−0.00579-0.00297	0.528	4.6	0.00022	−0.03173-0.03218	0.989	−1.1
Barrier to health care	−0.00007	−0.00032-0.00018	0.589	0.2	−0.00030	−0.00110-0.00049	0.454	1.5
Residential setting	0.00002	−0.00248-0.00252	0.986	−0.1	0.00104	−0.00161-0.00369	0.441	−5.1
Constant	-	-	-	-	−0.02333	−0.071840.02519	0.346	-

Coef., cluster-adjusted coefficients estimated by pooled Oaxaca-Blinder decomposition (including group indicator). *CI* Confidence interval. Pct., percentage attributable to individual predictor.

*LRR* Low-risk region, *HRR* High-risk region

**Table 6:** Summary decomposition of the gap in under-five mortality risk between LRR and HRR in Nigeria by specific predictors of under-five deaths.

	Explained component (Endowment - E)				Unexplained component (Coefficient - C)			
	Coef.	95% CI	*P*-value	Pct.	Coef.	95% CI	*P*-value	Pct.
Child’s sex	0.00005	−0.00006-0.00016	0.402	−0.2	0.00015	−0.00016-0.00046	0.339	−0.7
Child’s birth size	−0.00022	−0.00093-0.00049	0.544	0.7	−0.00682	−0.01317–0.00048	0.035	33.1
Child’s birth order	−0.00476	−0.00682–0.0027	0.000	15.5	0.00316	−0.00250-0.00881	0.274	−15.3
Child’s birth interval	−0.00155	−0.00234–0.00076	0.000	5.1	−0.02831	−0.04809–0.00853	0.005	137.5
Age at child’s birth	−0.00093	−0.00190-0.00004	0.061	3.0	0.01325	0.00105–0.02545	0.033	−64.3
Current marital status	0.00005	−0.00048-0.00058	0.847	−0.2	−0.00815	−0.03505-0.01875	0.553	39.6
Religious affiliation	−0.00507	−0.01164-0.0015	0.130	16.5	0.00078	−0.00766-0.00922	0.856	−3.8
Occupational category	0.00107	−0.00073-0.00288	0.245	−3.5	0.01381	−0.00315-0.03076	0.111	−67.0
Family planning status	−0.00384	−0.00623–0.00145	0.002	12.5	0.00329	−0.00170-0.00828	0.196	−16.0
Mass media exposure	0.00079	−0.00140-0.00298	0.482	−2.6	−0.00353	−0.00942-0.00237	0.241	17.1
Educational status	−0.00903	−0.01491–0.00314	0.003	29.4	0.00473	0.00000-0.00945	0.050	−22.9
Family structure	−0.00589	−0.00777–0.00401	0.000	19.2	0.00608	−0.00161-0.01377	0.121	−29.5
Household headship	−0.00106	−0.00234-0.00022	0.106	3.5	0.00704	−0.01378-0.02786	0.507	−34.2
Household wealth quintile	−0.00788	−0.01322–0.00254	0.004	25.7	−0.00181	−0.00509-0.00147	0.280	8.8
No. of household members	0.00885	0.00699–0.01071	0.000	−28.9	0.00088	−0.00609-0.00785	0.805	−4.3
Type of drinking water	−0.00025	−0.00094-0.00045	0.486	0.8	−0.00378	−0.00818-0.00062	0.092	18.4
Type of toilet facility	0.00045	−0.00062-0.00152	0.412	−1.5	0.00102	−0.00066-0.00270	0.236	−4.9
Type of cooking fuel	−0.00141	−0.00579-0.00297	0.528	4.6	0.00022	−0.03173-0.03218	0.989	−1.1
Barrier to health care	−0.00007	−0.00032-0.00018	0.589	0.2	−0.00030	−0.00110-0.00049	0.454	1.5
Residential setting	0.00002	−0.00248-0.00252	0.986	−0.1	0.00104	−0.00161-0.00369	0.441	−5.1
Constant	-	-	-	-	−0.02333	−0.071840.02519	0.346	-

Coef., cluster-adjusted coefficients estimated by pooled Oaxaca-Blinder decomposition (including group indicator). *CI* Confidence interval. Pct., percentage attributable to individual predictor.

*LRR* Low-risk region, *HRR* High-risk region

Additionally, the decomposition subsection of the table disaggregates the total inter-group gap (−0.05136) into its two-fold endowment part (−0.03084) and coefficient part (−0.02052). The findings suggest that the gaps in the mean prevalence of under-five mortality are mainly accounted for by the difference in the distribution of the predictors, with a 59.8% share of the total, rather than by differences in their effect, with a 40.2% share. In this case, the negative value of the endowment effect value indicates that if LRR and HRR had similar demographic and socioeconomic characteristics (e.g., the same distribution of birth order, education levels, household size, etc.), the overall gap in the risk of under-five mortality would decrease by about 3%-point or 60%. Similarly, the negative value of the coefficient effect indicates that if LRR and HRR had similar behavioural responses to under-five survival, the total risk difference would reduce by roughly 2%-point or 40%. These generally imply that the contexts in LRR are more favourable for reducing under-five mortality compared to those of HRR. The results of the detailed and expanded decomposition revealing the contributions of each observed determinant to both explained and unexplained components are contained in Tables 5 and 6, respectively.

Tables 5 and 6 convey the relative significance of the regressors with respect to their endowment and coefficient effects on under-five mortality risk differentials between LRR and HRR. Regarding the endowment part, the findings revealed that disparities in the distribution of child’s birth order and interval, mother’s family planning status and educational attainment, as well as family structure, household wealth status, and number of household members contributed significantly to the observed LRR-HRR gap in under-five mortality risks (*p* ≤ 0.05). Specifically, the aggregate results presented in Table 5 showed that the largest part of the disparity was explained by differences in mother’s educational status (29.4% share, *p* = 0.003), number of household members (−28.9% share, *p* = 0.000), household’s wealth quintile (25.7% share, *p* = 0.004), and family structure (19.2% share, *p* = 0.000). This set is followed in order of significance by mother’s family planning status (12.5% share, *p* = 0.002) and child’s birth order (15.5% share, *p* = 0.000). This implies that the compositions of the observed child, mother, and household’s attributes are protective and favour better survival of under-five children in the LRR compared to their HRR counterparts. Contrastingly, the results showed that the distribution of household membership size tends to widen the LRR-HRR gap in childhood survival significantly (*p* < 0.001). Mother’s age at child’s birth had a marginal contribution in explaining the LRR-HRR risk differential, accounting for only 3.0% of the total explained variance (*p* = 0.061).

Moreover, the expanded analysis presented in Table 6 elicited further decomposition of aggregate proportions of the explained variation attributed to the observed determinants into their constituent categories. For instance, the larger proportions of gap explained by mother’s educational status and household’s wealth quintile were respectively attributive to the magnitude of children born to mothers with primary education (14.6%-point of 29.4% share, *p* = 0.007) and secondary + education (15.1%-point of 29.4% share, *p* = 0.006), and children from households in the lower wealth quintile (12.6%-point of 25.7% share, *p* = 0.005) and upper wealth quintile (13.1%-point of 25.7% share, *p* = 0.011). Children from the LRR were at a considerable advantage in surviving beyond age 5 years based on the levels of these determinants as revealed by the results presented in Table 2. Concerning the coefficient component, the variable-by-variable analysis in Table 5 suggests that children from HRR are more predisposed to dying before reaching age 5 from the coefficient effects associated with birth size (33.1% share, *p* = 0.035) and birth interval (137.5% share, *p* = 0.005), but face lesser risks from the effects of age at child’s birth (−64.3% share, *p* = 0.033). Meanwhile, the results from the expanded analysis suggest, for instance, that under-five children in HRR suffer significantly greater mortality risks than those in LRR due to risks associated with first-order births and birth interval shorter than 36 months, whereas the protective effects of mother’s primary and secondary + education, and improved household wealth status tend to translate into better survival chances in the HRR than LRR.

## Discussion

This study quantified the distribution of U5M in Nigeria, exploring the gap in the determinants of childhood mortality between the country’s LRR and HRR. In this attempt, the study examined factors sustaining high U5M in HRR and those reducing such deaths across Nigeria’s LRR. Among the six geopolitical regions in the country, results from spatial analysis established almost all states in the North East and North West as hotspots for U5M. In the North West, U5M remained above the national average (132/1000 live births), with the least in Zamfara State (144/1000 live births). Similarly, in the North East, except for Borno State with 86 deaths per 1000 live births, the region has some of the states with the highest U5M in Nigeria. In contrast, U5M rates were relatively much lower in the North Central, South East, South South and South West regions compared to North East and North West, thus designating the former as coldspots for U5M in Nigeria. Similar North-South U5M differentials have been wholly^13,43^ or partly^10,18^ reported in the literature. Furthermore, considering the LRR-HRR dichotomy, this study established under-five mortality rates that ranged from 82 deaths per 1000 live births in the LRR to more than double this size (172 per 1000 live births) in the HRR. There are possibly unique practices, particularly about health-seeking behaviour, driving these outcomes^6^. Such findings stressed the need for not only the contextual assessment of U5M distribution across Nigerian regions but also the pursuit of targeted interventions for reducing U5M in the country.

Aligning with the findings from the descriptive analysis, results obtained from the Blinder-Oaxaca multivariate logistic regression decomposition established considerable inter-regional inequality in survival chances among under-five children in Nigeria. On average, the rate of dying among under-five children was approximately 59% higher in high-risk regions relative to low-risk regions. Empirical studies highlight significant regional variations in under-five mortality likelihoods in Nigeria, with more pronounced risks often observed among children born in the high-risk settings–the Northeastern and Northwestern regions–compared with their peers born elsewhere, especially the Southwestern region^10,17,18^. The aggregate decomposition further suggests that improving the demographic and socioeconomic conditions in the high-risk regions to the average levels observed in the low-risk regions has the potential to close the survival inequality/gap by roughly 60%. Estimates from national household surveys revealed stark demographic and socioeconomic disparities, with most states in northern Nigeria exhibiting poorer outcomes in education, living standards, and health^13,19,20^.

The study indicates that the gap in under-five mortality between the low-risk and high-risk regions is influenced by geographical disparities in child-, mother-, and household-level factors. Specifically, the differential composition of birth order, birth interval, family planning uptake, educational attainment, family structure, household wealth status, and household size contributed significantly to explaining the inter-regional variances in under-five mortality risks. Meanwhile, maternal education, household wealth status, and family size emerged as the strongest drivers. The findings corroborate evidence from past studies which examined inequalities in childhood deaths from various perspectives, including gender, education, wealth, place of residence, region, and time across countries^21–28^. The protective effects of high levels of maternal education and household wealth tend to accrue through increased awareness and implementation of health promotion strategies, and better access to healthy nutrition and sustained healthcare. For instance, poor access to health services and malnutrition have been identified as underlying causes of deaths among children, with strong links to parental education and household economic status^6,29–32^. This implies that interventions aimed at enhancing maternal education and increasing access to economic resources in the high-risk regions may prove effective for addressing the enduring regional disparities in childhood survival in Nigeria.

Furthermore, the relative contributions and significance of birth order, birth interval, and family planning uptake in explaining the observed mortality disparity underscore the need to expand access to low-cost, effective and culturally-acceptable contraceptive methods. Global initiatives promoting healthy timing and spacing of pregnancy and childbirth through family planning have been adjudged to be effective in reducing the incidence of high-risk pregnancies and childhood deaths, especially in low-resource settings^33–35^. High-risk fertility, comprising births within short intervals, after third-order birth, and in early/late reproductive years, has been found to elevate the likelihood of morbidity and mortality among children aged 0 to 59 months in different contexts^8,14,36^. Research indicates a strong inverse relationship between contraceptive prevalence and the proportion of high-risk pregnancies and childbirths in Nigeria and elsewhere^37,38^. Coincidentally, modern contraceptive prevalence is lowest in the regions considered high-risk for child deaths at about 6–8% compared to 14–24% in the low-risk regions, whereas high-risk birth rates vary contrariwise^9,13^. Projections based on DHS data from low- and middle-income countries suggest that around 50–70% of maternal and child deaths could be avoided if family planning programs could meet the need for contraception among women predisposed to high-risk births^39^.

### Strengths and limitations

In-country context assessment has been suggested to proffer a better realistic understanding of U5M by multi-country studies^21,44^, emphasising the unique realities of each country, this study was conducted within Nigeria, quantifying LRR-HRR gaps in the determinants of U5M. Although the Oaxaca-Blinder decomposition approach had been employed in a multi-country study to determine U5M, such was based on the rural-urban dichotomy^44^, whereas the current study adds to the existing literature by undertaking decomposition analysis on the core interest of the low-high dichotomy of under-5 deaths in Nigeria. The study is not without its limitations. Reports of under-five deaths in the NDHS were adequate to the extent of mothers’ recall capacity and willingness to provide such information. This holds potential for recall bias on both death and related information. This study adopted a quantitative approach in its analysis to determine U5M determinants, viz-a-viz LRR-HRR differentials. Future studies should explore qualitative methods to probe particularly identified important correlates and other covariates outside of those explored in this study. This will provide a deeper understanding and peculiar patterns of U5M trends in each region.

## Conclusion

The study established a more than two-fold higher under-five mortality rate for the HRR compared to the LLR. The findings suggest that improving the health and demographic factors (birth order, birth interval, family structure, household size, family planning use) and socioeconomic conditions (educational attainment, household wealth status) in the high-risk regions to the levels observed in the low-risk regions has the potential to close the existing inequality/gap in child survival between the HRR and LLR of Nigeria. The study concludes that interventions aimed at improving women’s demographic and socio-economic situations as well as increasing access to healthcare services in the high-risk regions are fundamental to addressing the enduring regional disparities in childhood survival in Nigeria.

## Data Availability

The data used for this study is available at http://dhsprogram.com.
